# Proteomic analysis highlights the role of detoxification pathways in increased tolerance to Huanglongbing disease

**DOI:** 10.1186/s12870-016-0858-5

**Published:** 2016-07-28

**Authors:** Federico Martinelli, Russell L. Reagan, David Dolan, Veronica Fileccia, Abhaya M. Dandekar

**Affiliations:** 1 Department of Agricultural and Forest Sciences, University of Palermo, viale delle scienze ed. 4, 90128 Palermo, Italy; 2 Plant Sciences Department, University of California, One Shields Avenue, 95616 Davis, CA USA

**Keywords:** *Citrus*, Huanglongbing, *Candidatus liberibacter*, iTRAQ, Proteome, Proteomic

## Abstract

**Background:**

Huanglongbing (HLB) disease is still the greatest threat to citriculture worldwide. Although there is not any resistance source in the *Citrus* germplasm, a certain level of moderated tolerance is present. A large-scale analysis of proteomic responses of *Citrus* may help: 1) clarifying physiological and molecular effects of disease progression, 2) validating previous data at transcriptomic level, and 3) identifying biomarkers for development of early diagnostics, short-term therapeutics and long-term genetic resistance.

**Results:**

In this work we have conducted a proteomic analysis of mature leaves of two *Citrus* genotypes with well-known differing tolerances to HLB: Navel orange (highly susceptible) and Volkameriana (moderately tolerant). Pathway enrichment analysis showed that amino acid degradation processes occurred to a larger degree in the Navel orange. No clear differences between the two genotypes were observed for primary metabolic pathways. The most important finding was that four glutathione-S-transferases were upregulated in Volkameriana and not in Navel orange. These proteins are involved in radical ion detoxification.

**Conclusions:**

Upregulation of proteins involved in radical ion detoxification should be considered as an important mechanism of increased tolerance to HLB.

**Electronic supplementary material:**

The online version of this article (doi:10.1186/s12870-016-0858-5) contains supplementary material, which is available to authorized users.

## Background

Huanglongbing disease currently threatens areas where *Citrus* cultivation is important in the agricultural economy such as East Asia, the Middle East, and the Americas. Huanglongbing disease is caused by three species of *Candidatus* liberibacter asiaticus (CaLas), americanus and africanus [1]. The pathogen is transmitted by two species of psyllids: Diaphorina citri and Trioza erytreae. Recently, Trioza erytreae was found for the first time in Europe (Galicia, Spain). Typical symptoms of Huanglongbing disease in leaves include shoot yellowing and blotchy, mottled leaves. Although most of the fruits are still of commercial quality, fruits from severely affected branches are unmarketable: small, lopsided, green, and acidic, with many aborted seeds. Leaves accumulate starch, phloem is damaged and cell wall lamellae swell during CaLas infection [1, 2]. *Candidatus liberibacter* spp. belong to the alpha subdivision of the proteobacteria based on ribosomal region sequence data [3]. The bacterium has not yet been definitively cultured despite attempts to do so [4]. Koch’s postulates have not been fulfilled for this disease, so possible interactions with other microrganisms cannot be ruled out. The pathogen lives in the insect and in the phloem of *Citrus* trees. Once acquired, it typically persists for the rest of the life of the host. Insecticides can decrease psyllid populations, but since the pathogen remains in the vector, disease spread can occur with the presence of just a few infected psyllids in the orchard. All genotypes within the genus *Citrus* are susceptible to HLB to varying degrees although species of other close-related genera showed some sort of resistance [5]. There is variability in disease severity and symptoms among *Citrus* genotypes [6]. *Murraya paniculata* (orange jasmine), an ornamental *Citrus* closely-related plant, showed fewer symptoms of the disease [7]. A study examining the responses of 30 genotypes to HLB disease grouped them based on phenotypic analysis of induced symptoms [5]. Another recent study has evaluated 65 *Citrus* accessions and 33 accessions belonging to other closely related genera. Resistance was reported in accessions not belonging to *Citrus* genera [8]. Another work have screened Citrus germplasm susceptibility to HLB analyzing sixteen Citrus genotypes [9]. Results showed that *Citrus macrophylla* and *C. medica* were the most susceptible while complex genetic hybrids ‘US 1-4-59’ and ‘Fallglo’ were the least susceptible. A metabolomic investigation was conducted comparing five different tolerant hybrids and a highly susceptible cultivar to identify potential metabolites linked with diverse response [10]. The causes of the disease have been studied using different “omic” approaches to identify which genes, proteins and metabolites may be targeted by innovative diagnostic and therapeutic methods. The genome of the pathogen was sequenced using a metagenomic approach, both from infected plants [11] and the insect vector [12]. No toxins or other secreted proteins have been linked to the disease and the mechanisms of its pathology are still unclear. Large scale microarray analysis revealed significant modulation of genes involved in transport, cell defense and carbohydrate metabolism [13, 14]. Photosynthesis is diminished in both young and mature leaves, but it is upregulated in infected fruits [14]. Starch accumulation was linked to the upregulation of genes involved in glucose import into the chloroplast and starch biosynthesis [15, 16]. A modulated Jasmonic (JA)-Salicilic acid (SA) crosstalk of innate responses may lead to a misdirected defence response. An integrated approach of 2-DE and mass spectrometry showed that changes in levels of several proteins involved in photosynthesis and protein synthesis were linked to reduced concentrations of Ca, Mg, Fe, Zn, Mn and Cu in infected grapefruit leaves [17]. Proteins upregulated in infected samples were involved in redox stage and cell defense such as Cu/Zn superoxide dismutase, peroxidases, chitinases and lectin-related proteins [18]. ‘Madam Vinous’ sweet orange plants infected by CaLas showed increased miraculin-like proteins, chitinase, Cu/Zn superoxide dismutase and lipoxygenase. Some key metabolites modulated by HLB include proline, β-elemene, (−)-trans-caryophyllene, and α-humulene [19]. Increased accumulation of some amino acids (L-proline, L-serine, and L-aspartic acid) and organic acids was linked to greater susceptibility of ‘Madam Vinous’ sweet orange compared to Carrizo citrange [20]. However, it is worthy to notice that these trees were highly infected with many secondary effects so it will be necessary to confirm these results with newly infected trees. An increased amount of most amino acids, involved in plant defense to pathogens was observed in tolerant varieties in such as phenylalanine, tyrosine, tryptophan, lysine, and asparagine [21].

This study examines proteomic changes in fully photosynthesizing leaves to determine how disease mechanisms and susceptibility vary between two *Citrus* genotypes, using an integrated approach of principal component analysis (PCA), gene set and pathway enrichment analysis. The purpose was to characterize key proteins and post-transcriptionally modulated pathways in different *Citrus* genotypes at a late symptomatic stage of HLB.

## Results and discussion

Navel orange (*Citrus* sinensis (L.)) is an HLB-sensitive cultivar while Volkameriana is moderately tolerant [5]. Different techniques (2-DE, mass spectrometry and ICP mass spectroscopy) have been used to identify key proteins differentially regulated by HLB in *Citrus* leaves [19, 20]. Over 4000 proteins were analyzed using isobaric tags for relative and absolute quantitation (iTRAQ) for both genotypes (4557 in Volkameriana and 4521 in Navel orange). In Navel orange, 599 proteins were differentially regulated between infected and healthy tissue (P-value < 0.05 and Log_2_ FD > 0.5 and < −0.5) (Additional file 1: Table S1). In Volkameriana, 411 differentially regulated proteins were found between infected and healthy tissue (Additional file 2: Table S2).

### PCA analysis

**Fig. 1:**
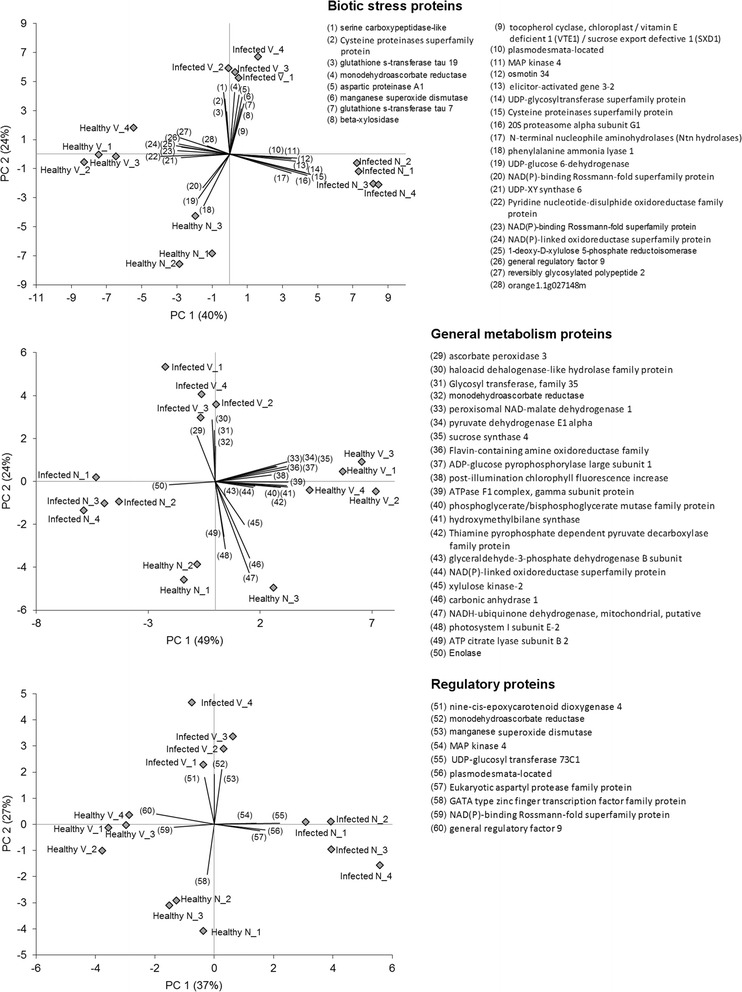
Principal component analysis of differentially regulated proteins of four types of leaf tissues (Control Navel orange, Control Volkameriana, Infected Volkameriana and Infected Navel orange). Proteins that contribute highly to the separation of the the four samples are numbered and listed next to each graph

PCA was used to visualize differences between the four analyzed genotypes x disease status, subdividing the entire proteomic profile into three important subcategories: biotic stress responses, overall cell metabolism and transcriptional regulation pathways (Fig. 1). All identified proteins belonging to these important functional categories were used for the PCA plots. The four categories of samples (healthy V, healthy N, infected V and infected N) were clearly separated in all three PCA plots, implying significant protein changes in all three gene categories related to species and health status. For biotic stress-related proteins, PC1 and PC2 accounted for 40 and 24 % of the data variability, respectively. Important proteins associated with each functional category that contributed to separation between sample types (indicated by directions of vectors) are listed in Fig. 1. Some key proteins involved in redox state significantly contributed in the separation of Infected V from the rest of the other categories. In the general metabolism PCA plot, the PC1 and PC2 accounted for 49 and 24 % of the data variability, respectively. Key proteins involved in primary metabolism associated with the separation of Healthy V from the Infected V include malate dehydrogenase and pyruvate dehydrogenase (TCA cycle-glycolysis), sucrose synthase and AGPase (sucrose and starch metabolism). The third PCA plot was generated based on the expression of proteins involved in transcriptional regulation, signaling, hormone and redox state. PC1 and PC2 accounted for 37 and 27 % of data variability, respectively. Interestingly, the regulation of few important proteins seems to specifically characterize the infected vs. healthy state of Navel orange. These include MAP kinase 4, UDP-glucosyl transferase, and aspartyl protease. No changes were observed for MAPK6. Proteins that appear in the three PCA plots were highly regulated in the comparison between infected and control in both *Citrus* genotypes. Indeed, they may be considered as putative candidate biomarkers of a clear symptomatic status in *Citrus* at proteomic level. Their HLB-regulated pattern of expression greatly contributed in distinguishing the four different leaf sample types. Further analysis will be needed to validate these data and confirm the role in the pathogenesis of HLB disease.

### Gene set and pathway enrichment analysis

**Fig. 2:**
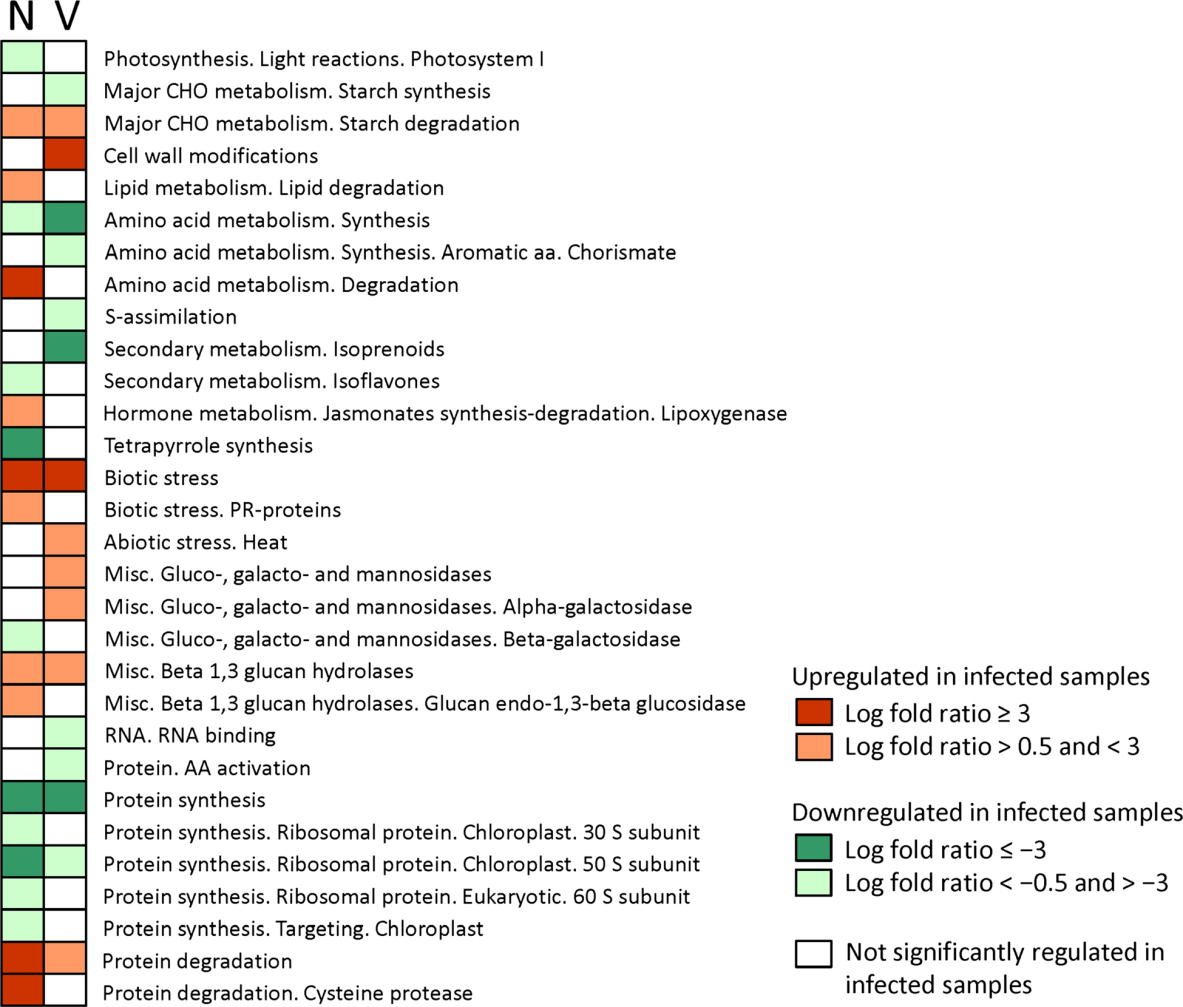
Gene set enrichment analysis using Pageman web-tool. Upregulated and downregulated pathways at proteomic level in infected Volkameriana (V) and infected Navel orange (N) in comparison to respective healthy controls

The Pageman web tool highlighted which gene categories were up- or down-regulated in each pairwise comparison (Fig. 2). Both infected genotypes exhibited repressed amino acid biosynthesis and protein synthesis. In Navel orange, photosynthesis, isoflavone pathways, tetrapyrrole synthesis, and galactose metabolism were significantly inhibited by HLB. In Volkameriana, S-assimilation, isoprenoids, RNA binding and amino acid activation were specifically diminished. In both genotypes, HLB enhanced starch-related pathways, biotic stress-related proteins, beta 1,3 glucan hydrolases, and protein degradation pathways. Some distinct differences were observed between genotypes in HLB response. In Volkameriana, cell wall modifications, galactose metabolism, and heat shock proteins were upregulated. In Navel orange, amino acid degradation, lipid metabolism, jasmonates, and PR-proteins were upregulated.

A pathway enrichment analysis was performed using the DAVID bioinformatic resource to determine which metabolic pathways were commonly or specifically affected by HLB disease in both genotypes (Table 1). Some metabolic pathways were altered by HLB in both species. Amino acid metabolism (glycine, serine, threonine, phenylalanine and tryptophan) was significantly downregulated. Other key inhibited pathways include biosynthesis of plant hormones, terpenoids, and phenylpropanoids. On the other hand, tyrosine metabolism was upregulated in both genotypes. In Navel orange, fatty acid biosynthesis and nitrogen metabolism were diminished while alpha-linolenic acid metabolism was enhanced. In Volkameriana, alkaloids and pyruvate metabolism-related proteins were repressed while galactose metabolism and fatty acid metabolism were upregulated.

**Table 1:** Pathway enrichment analysis using DAVID Bioinformatics Resources 6.7. Pathways that were upregulated and downregulated for the healthy/infected comparison for each genotype are indicated with the corresponding *p*-value

Pathway	Navel orange		Volkameriana	
	Up	Down	Up	Down
Fatty acid biosynthesis		9.2*10^−3^		
Glycine, serine and threonine metabolism		1.3*10^−2^		2.6*10^−2^
Biosynthesis of plant hormones		1.5*10^−2^		1.4*10^−3^
Porphyrin and chlorophyll metabolism		1.6*10^−2^		2.9*10^−2^
Phenyalanine, tyrosine and tryptophan biosynthesis		2.7*10^−2^		3.3*10^−3^
Carbon fixation in photosynthetic organisms		2.9*10^−2^		
Terpenoid backbone biosynthesis		3.0*10^−2^		2.1*10^−2^
Nitrogen metabolism		3.3*10^−2^		
Biosynthesis of phenylpropanoids		4.8*10^−2^		2.3*10^−2^
alpha-linolenic acid metabolism	1.6*10^−3^			
Tyrosine metabolism	8.3*10^−3^		1.8*10^−2^	
Biosynthesis of alkaloids from shikimate pathway				4.4*10^−3^
Pyruvate metabolism				2.5*10^−2^
Biosynthesis of alkaloids from terpenoid and polyketide				4.5*10^−2^
Proteasome			2.7*10^−3^	
Galactose metabolism			8.2*10^−3^	
Fatty acid metabolism			4.3*10^−2^	

### Primary metabolism

The integration of the two pairwise comparisons into a unified Mapman visualization allowed us to identify proteins that were commonly or specifically regulated in response to HLB in the two species. Some proteins involved in cell wall modifications were upregulated by HLB only in Volkameriana: expansin A1, expansin A8, expansin-like B1, xyloglucan endotransglycosylase 6, and xyloglucanxyloglucosyltransferase (TCH4) (Additional file 3: Figure S1). In Navel orange, proteins involved in fatty acid biosynthesis and elongation were generally repressed while several key proteins involved in amino acid degradation were upregulated: arginase, pyrroline-5-carboxylase reductase, lactoylglutathione lyase, and 3-hydroxylmethylglutaryl-CoA lyase. The increase of protein degradation indicates that senescence processes may be more highly activated in HLB-diseased Navel orange than in Volkameriana.

**Fig. 3:**
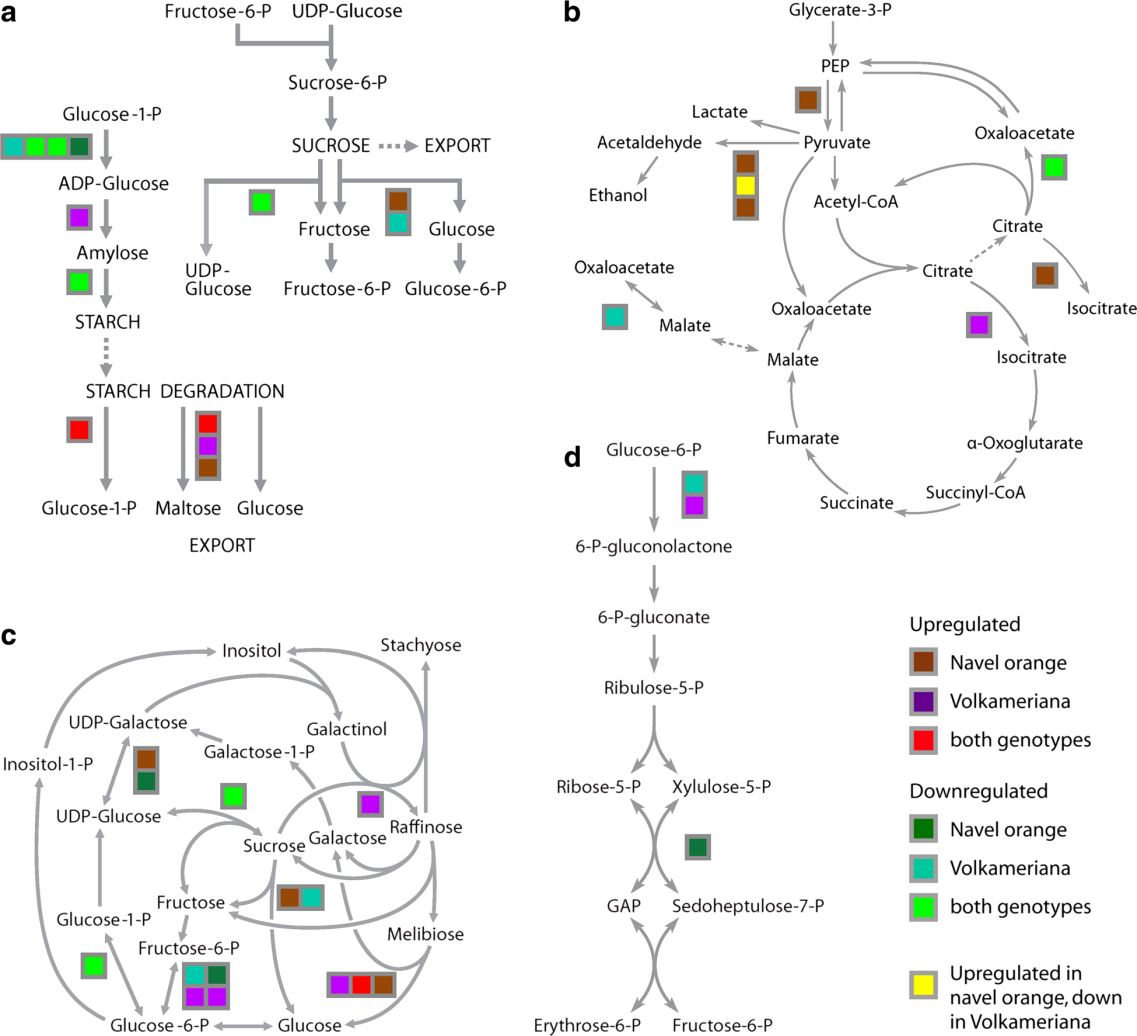
Proteins involved in primary metabolic responses to HLB. A) Sucrose and starch metabolism; B) Raffinose metabolism; C) TCA cycle; D) Oxidative pentose phosphate. Proteins that were differentially expressed between control and infected trees are indicated by colors, based on their pattern of expression in the two genotypes (see color key). Each colored square represents expression change in a protein catalyzing a step in the pathway. More than one square grouped together indicates different members of the same protein family found to be differentially regulated

Sucrose metabolism was only slightly affected by HLB at the protein level; only sucrose synthase was repressed. Starch metabolism was more altered (Fig. 3a). The first enzyme of starch biosynthesis, ADP-glucose pyrophosphorylase, was inhibited by HLB in both genotypes. In Volkameriana, starch synthase was upregulated while 1,4-alpha-glucan starch branching enzyme was slightly downregulated. Among starch degradation enzymes, glucan phosphorylase and heterogycan glucosidase 1 were upregulated in both genotypes. Alpha-amylase was upregulated in Volkameriana while beta-amylase 6 was enhanced in Navel orange. Taken together these findings showed that starch metabolism was highly affected in both genotypes at the protein level. No clear association between differing susceptibility to HLB is evident from starch pathway regulation alone.

A significant downregulation in HLB-infected samples was observed for proteins involved in photosynthetic reactions [17]. The altered transcription of sugar and starch metabolism genes caused by HLB [16] mostly agreed with the corresponding protein changes presented in the present work. The observed changes in starch-related pathways were consistent with the transcriptomic analysis [16]. Increased starch degradation was observed probably due to the increased starch concentrations in infected leaf tissues. ADP glucose-pyrophospholyase (ADPase) was repressed in both infected species. This enzyme is rate-limiting for starch biosynthesis, catalyzing the conversion of glucose-1-phosphate to ADP-glucose that is polymerized into amylopectin or amylose [22]. Starch accumulation in infected leaves is a typical symptom of HLB [1]. However, the greatest occurrence of this process may occur at an early, asymptomatic stage. Indeed we may speculate that the accumulation of starch may be a secondary effect of the disease instead of being the cause of symptoms. When symptoms are already evident and yellowing is present, starch biosynthesis may slow down and starch degradation is expected to be activated as a response of the plant to limit damage to cell structures. However, some differences in primary metabolism were observed between transcriptomic and proteomic approaches. Changes in invertase gene expression were observed [16], but not its protein levels. Sucrose, the substrate of invertase, is produced by photosynthesis in leaves and then transported to sink tissues (immature fruits and young leaves) through the phloem. Involvement of sucrose in signaling of innate responses has been described recently [23]. Invertase plays an important role in plant stress responses, possibly serving as an extracellular signal of pathogen attacks [24]. It is possible that expression of this gene in response to HLB depends on genotype, plant physiological conditions and age, tissue developmental conditions, type of infection (psyllid inoculation or graft-mediated) and/or environmental conditions (field or controlled environment). Taken together all these primary metabolism results concur with previous findings showing significant modification of transcript abundance in minor carbohydrate metabolism [16] but do not suggest a clear link to the well-known difference in tolerance between the two genotypes.

GPT2 has been linked with HLB disease: this gene is responsible for glucose import into the chloroplast and consequently for starch accumulation [13, 16]. The protein was not found among those extracted and characterized by iTRAQ, therefore no conclusions can be made about changes in protein levels due to HLB. However, the protocol used to analyze the *Citrus* proteome favored detection of soluble cell proteins over membrane proteins such as GPT2.

Expression of key proteins involved in the TCA cycle and PEP metabolism were affected by HLB. ATP-citrate lyase subunit B2 was repressed in both species (Fig. 3b). Alcohol dehydrogenase 1 which converts aceltaldehyde to ethanol, was upregulated in both genotypes. In infected Navel orange, isopropyl malate isomerase, which converts citrate to isocitrate and pyruvate decarboxylase involved in fermentation, was more abundant than in healthy tissue. In Volkameriana, pyruvate decarboxylase was repressed while aconitate hydratase involved in TCA cycle was upregulated.

Raffinose metabolism was drastically altered by the disease (Fig. 3c). In Navel orange, expression of alpha-galactosidase 1, UDP-glucose-4-epimerase, glucose-6-phosphate isomerase, and alpha-galctosidase 1 were enhanced by HLB. In Volkameriana, raffinose synthase, phosphofructokinase 3 and phosphoglucomutase were upregulated. Sucrose synthase 4 and phosphoglycerate mutase were donwregulated in both genotypes. A transketolase that converts the xylulose-5-P in sedoheptulose-7-P was repressed in infected Navel orange (Fig. 3d).

Significant repression of aspartate biosynthesis and serine metabolism was observed in infected Navel orange leaves. This agrees with a previously described downregulation of serine-type peptidases at both asymptomatic and symptomatic stages [18]. In infected Navel orange, seventeen proteins involved in amino acid biosynthesis were downregulated. Fewer proteins were downregulated in infected Volkameriana. On the other hand, some upregulated proteins involved in amino acid degradation were identified only in infected Navel orange. Taken together, these findings suggest that amino acid metabolism in Navel orange is more sensitive to degradation during HLB infection than in Volkameriana.

### Secondary metabolism

A general repression of key proteins involved in biosynthesis of secondary metabolites was observed in both cultivars in response to CaLas infection (Additional file 4: Figure S2). Geranylgeranyl reductase and 1-deoxy-D-xylulose 5-phosphate reductoisomerase, involved in the non-MVA pathway, were repressed. Other commonly HLB-downregulated proteins involved in the shikimate pathway included 2-dehydro-3-deoxyphosphoheptonate aldolase, 3-dehydroquinate synthase, 3-phosphoshikimate-1-carboxylvinyltransferase and mevalonate diphosphate decarboxylase. Two key proteins involved in phenylpropanoids, phenylalanine ammonia lyase and aryl-alcohol dehydrogenase, were more abundant in both species in response to HLB, while cinnamyl alcohol dehydrogenase 9 was repressed. Two proteins involved in alkaloid biosynthesis, tropinone reductase and strictosidine synthase-like 4, were upregulated in infected Volkameriana.

**Fig. 4:**
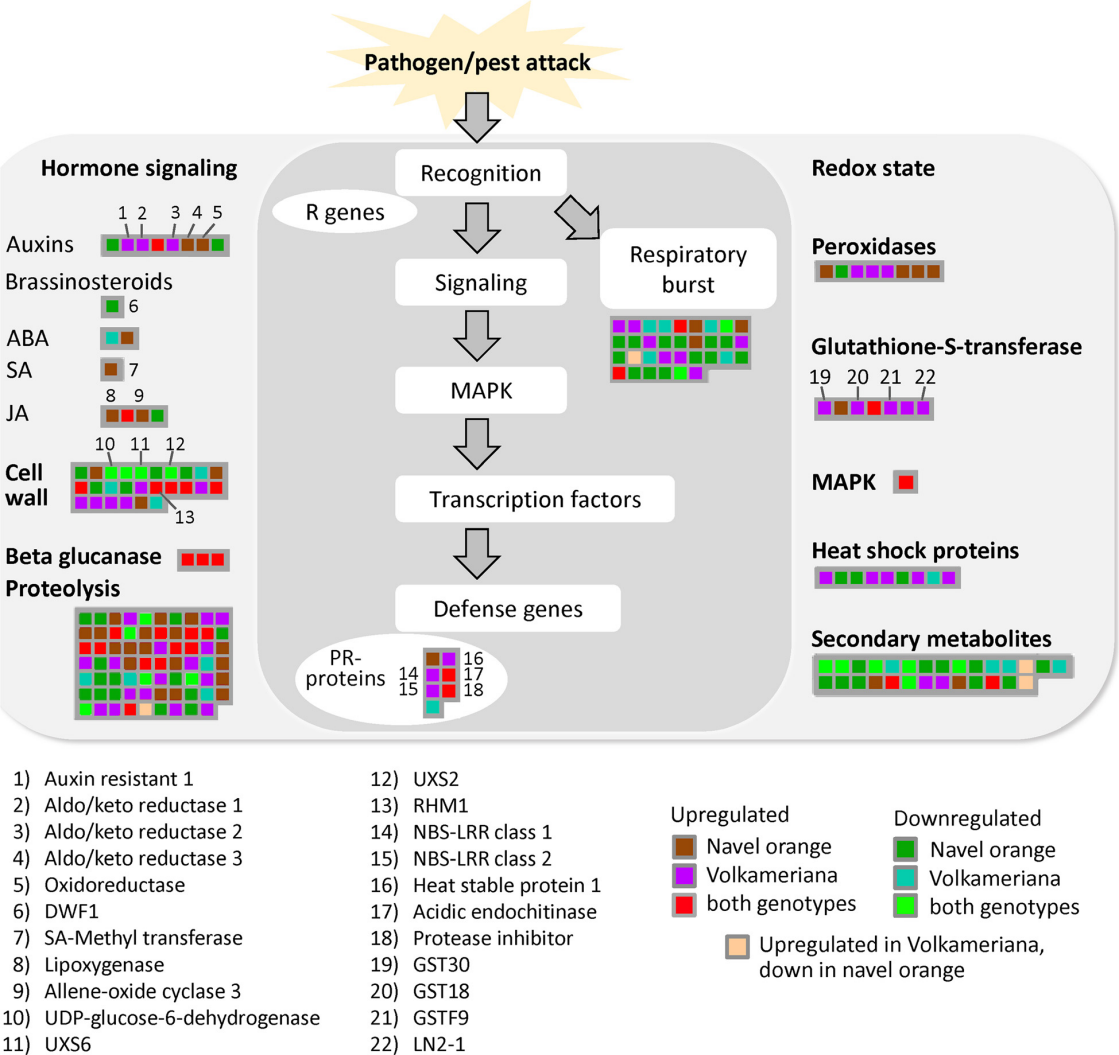
Biotic stress–related proteins altered in response to HLB disease in both Citrus genotypes. Proteins that were differentially expressed between control and infected trees are indicated by colors, based on their pattern of expression in the two genotypes (see color key). Each colored square represents expression change in a protein associated with regulatory and enzymatic functions

Findings related to *Citrus* activated defense responses against CaLas infection are shown in Fig. 4). Three proteins involved in auxin signal transduction were activated in infected Volkameriana: auxin resistant 1 and two aldo/keto reductases. One aldo/keto reductase was HLB-regulated in both genotypes. Three proteins involved in jasmonic and salicylic acid responses were induced in Navel orange but not in Volkameriana. Lipoxygenase 2 was upregulated in both genotypes.. Some key proteins involved in cell wall modifications were commonly regulated by both genotypes: UDP-glucose-6-dehydrogenase, UXS6, UXS2, and RHM1. Proteolytic-related proteins were altered in both genotypes. Taken together these findings do not suggest any clear association between the two genotypes and proteomic changes in hormonal crosstalk, cell wall and proteolytic pathways.

**Fig. 5:**
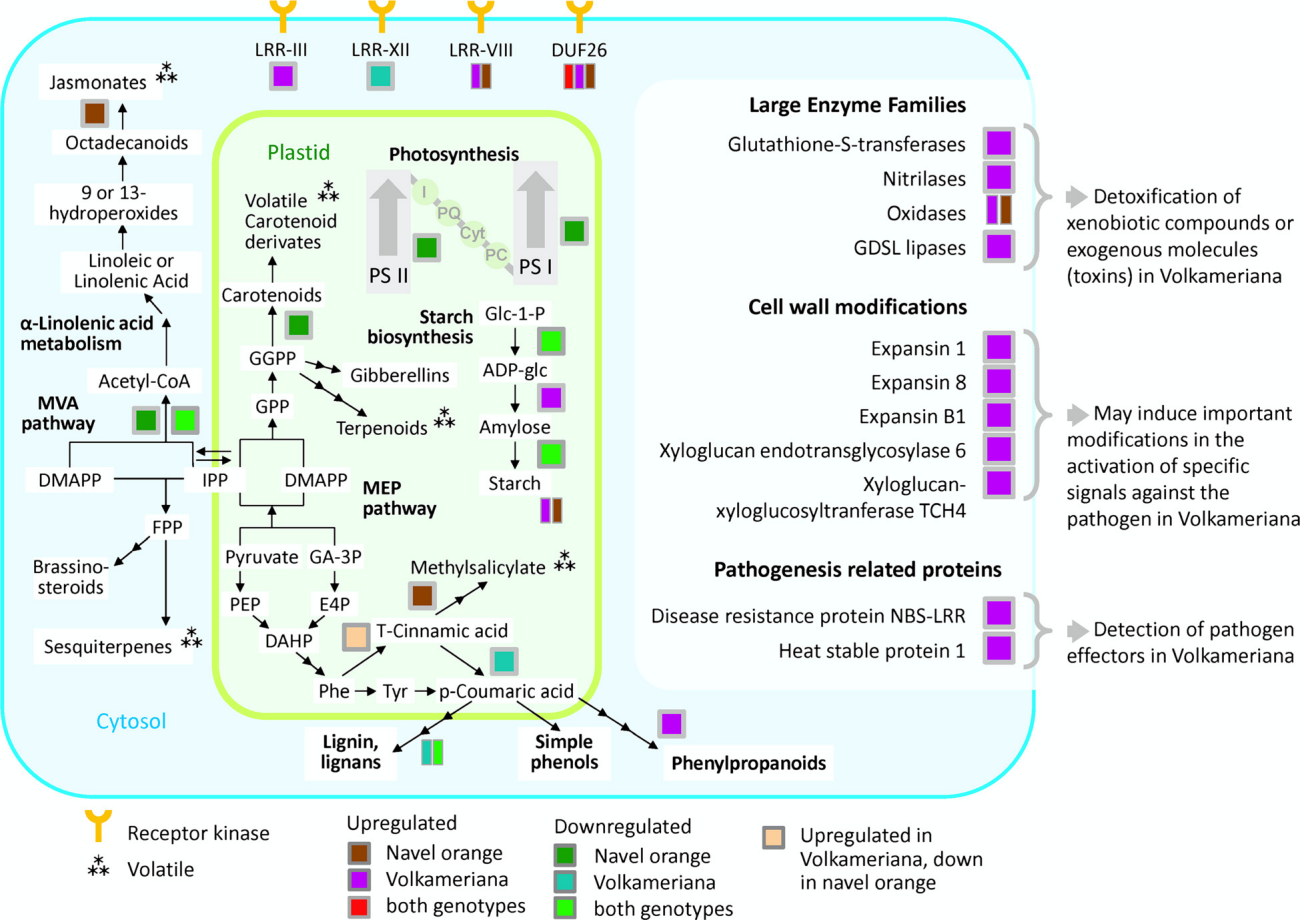
Global view of proteomic changes in Citrus leaves (Volkameriana and Navel orange) in response to CaLas infections. Proteins, pathways, and cell functions that were differentially expressed are indicated by colors, based on their pattern of expression in the two genotypes. Each colored square represents expression change in a protein associated with regulatory and enzymatic functions. More than one rectangle grouped together indicates different members of the same protein family were found to be differentially regulated

Some proteins synthesizing volatiles via the phenylpropanoid and carotenoid pathways were affected in the present study (Fig. 5). The marked differences between the two species suggests that to be effective, any innovative HLB-detection system based on induced volatiles must be cultivar-specific.

Plant phenols not only counteract reactive oxygen species and pathogen-secreted toxins, but also play roles in transport and signal transduction pathways. Polyphenol chemistry is critical to adapting plants to environmental stresses, including pathogens [25]. Phenylalanine ammonia-lyase (PAL) was downregulated by HLB infection in both species (Additional file 4: Figure S2). This enzyme is a key regulatory point for the entire phenylpropanoid pathway. Enhancement of its transcript abundance is linked to phytopathogen attacks [26]. Another important protein encoding isoflavone reductase and involved in antioxidant reactions was repressed in both the infected species, consistent with previous proteomic analysis [18]. In general, RNA-seq analysis showed that phenylpropanoid pathways were transcriptionally affected by HLB in both young and mature infected leaves in field grown mature trees [16]. These previous findings are not completely consistent with the present proteomic data. Gene set enrichment analysis showed a general downregulation of secondary metabolism in both genotypes, although some key proteins were upregulated in response to the disease. These contrasting findings may reflect differences in developmental and physiological stages of the plants analyzed in the two studies or differences in environmental and agronomic conditions. Although phenylpropanoids may be activated at early stages of infection, their repression when symptoms are severe is expected. The large number and complexity of metabolites belonging to phenylpropanoid pathways makes the clarification of their many roles in the host-pathogen battle difficult.

### Hormonal crosstalk

Plant innate responses are finely controlled by hormonal crosstalk, particularly between JA and SA signaling. The induction of allene oxide cyclase in infected Navel orange (Fig. 4) is intriguing because jasmonic acid signal transduction is a key pathway activated in response to necrotroph and herbivore attacks. JA-responsive proteins, gibberellin signaling (GASA1 and gibberellin-2-oxydase), and auxin signaling (CYP711A1 and SAUR-like proteins) were upregulated in mature infected leaves of Valencia orange [16]. Because CaLas is a biotroph, the activation of JA may be deleterious for the host as previously suggested [16]. Brassinosteroids affect disease resistance in plants [27] probably due to the induction of BAK1, which interacts with PAMP receptors such as bacterial flagellin to activate immune responses [28]. The ST1 gene was repressed in HLB-infected leaves [16]. However, the connections of brassinosteroids with the SA-ET-JA crosstalk and plant immunity remain elusive. On the other hand, auxin-related proteins were upregulated by HLB in both *Citrus* genotypes. Because of the antagonist role of auxins toward SA response [29], we may speculate that these effects are deleterious to the infected host.

Proteomic studies have revealed post-transcriptional regulation of genes involved in key pathways which may be responsible for variations in phenotypic responses to HLB. The regulation of carbohydrate metabolism (sucrose, starch, and raffinose metabolism) is clearly altered at symptomatic stage at both transcript and protein levels. Differences in signaling mechanisms and hormone-mediated defense responses may also contribute to the range of tolerance to HLB within the *Citrus* germplasm. The most compelling finding was that proteins involved in redox pathways and defense against xenobiotics (especially GSTS) were more abundant in Volkameriana than in Navel orange, and this may be linked with the former’s greater tolerance. Four GSTs were significantly upregulated in infected Volkameriana and not in infected Navel orange: GST18, GST19, GHST30, LN2-1. These effects on the proteome may explain the greater susceptibility of Navel orange compared to Volkameriana. While these findings regarding *Citrus* responses are valuable in the ongoing efforts to combat this deadly disease, further studies are still needed to validate these findings and deliver effective targets to develop new therapeutic strategies.

### Signaling and defense response pathways

Some proteins encoding receptor kinases of the LRR type were HLB-regulated: one was upregulated in Volkameriana, one was repressed in Navel orange and two different proteins of LRR-VIII were upregulated (one in Volkameriana and one in Navel orange). Leucine-rich repeat receptor kinases are the largest category of receptor kinases and mediate signaling of defense responses in plants. Other receptor kinases (VIII and DUF26) were upregulated in both *Citrus* genotypes. The proteins belonging to Domain of Unknown Function 26 were upregulated in response to HLB: one in Volkameriana, one in Navel orange and one commonly regulated. DUF26 is one of the largest classes of receptor-like kinases (RLKs). These proteins play important roles in regulating pathogen defense and programmed cell death [30]. Based on the proteomic results in this study, we speculate that diverse signaling mechanisms occur depending on the *Citrus* genotype. It is possible that variability in susceptibility of *Citrus* may result from pathogen perception due to activation of different receptors which in turn activate defense responses which vary in speed and intensity. Much remains to be learned regarding which receptor family is involved in susceptible or resistant responses to HLB.

Several proteins involved in calcium regulation were less abundant in infected leaves of Navel orange: calmodulin-binding and calcium-transporting ATPase. This is consistent with the significant drop in calcium concentration observed in symptomatic leaves [17]. Three 14-3-3 proteins were also HLB-downregulated in Navel orange and not in Volkameriana. These are a large family of proteins present in all eukaryotic organisms that aid signaling by binding other proteins such as kinases, phosphatases, and receptors (i.e. the P-type H+ ATPases) [31].

Volkameriana showed significant stimulation of respiratory burst and consequent redox state, a prerequisite of the upregulation of pathogenesis-related proteins. Enzymes involved in the control of reactive oxygen species were generally enhanced in response to CaLas infection in both genotypes although Volkameriana showed a higher activation of glutathione-S-transferases (GST30, GST18, GSTF9, LN2-1). The upregulation of these important detoxification proteins may be linked with the increased tolerance of Volkameriana in comparison to Navel orange. An upregulation of enzymes involved in the biosynthesis of peroxiredoxins, Cu/Zn superoxide dismutase and 2Fe-2S ferredoxin-like protein, occurred at both asymptomatic and symptomatic stages [18]. Glutathione S-transferase family proteins include several isozymes that help detoxify xenobiotic compounds [32]. Plant GSTs add glutathione to electrophilic xenobiotic molecules pushing them into the cell vacuole. Regulation of these proteins by environmental stress stimuli suggests a role in protection against any harmful event [33]. GSTs have been linked with hormone homeostasis and their high affinity for auxins suggests their upregulation is a general signal of responses to stress [34]. Stress-inducible GSTs conjugate deleterious metabolites caused by oxidative damage. Inducible GSTs may play the important role of detoxifying exogenous molecules such as phytotoxins produced by pathogen attacks. Higher levels of GSTs in Volkameriana strengthens the hypothesis that they protect against dangerous molecules generated by CaLas attack. Indeed, differential activation of GSTs may explain some of the variability of *Citrus* responses to HLB. Some peroxidases were also upregulated in both genotypes. These enzymes detoxify excess H_2_O_2_ [35] and they are grouped based on their subcellular localization [36]. As previously suggested [37, 174], greater *Citrus* susceptibility to HLB may be linked to a failure to rapidly induce antioxidant components to alleviate the devastating effects of ROS produced by CaLas. It has been suggested [38] that this category of proteins may be considered candidate markers in field-grown for the detection of the devastating disease “Esca” in grapevine. In the same way high expression of GSTs are potential candidate markers for genotypes with useful tolerance to HLB. Further investigations will need to be conducted for a large number of genotypes.

Pathogenesis-related (PR) proteins are plant defensive proteins against biotic attacks [39]. More PR-proteins were induced in Volkameriana than in Navel orange, consistent with the differing tolerance. Resistance (R) genes specifically activate a resistance reaction to a particular pathogen. NBS-LRR proteins are the most numerous R-gene class. NBS-LRR genes are finely controlled by regulatory mechanisms that allow their expression only when a biotic attack occurs, and limiting their metabolic cost when they are not required [40]. It is possible that the two NBS-LRR proteins upregulated in Volkameriana may contribute to enhanced tolerance of HLB disease. Heat shock proteins (HSP) are molecular chaperones with important functions in non-covalent protein folding or unfolding, assembly, and modifications. Genes encoding HSP70, HSP82 and other small heat shock proteins were expressed at lower levels in HLB disease in both fruit and leaf tissues [16, 41]. Down-regulation of HSP70, chaperon-60kD and chaperonin-60alpha was also seen in infected grapefruit [17]. In the present study, HSP81, HSP21 and HSP23 were induced in Volkameriana while several HSP proteins were inhibited in Navel orange. Taken together, we conclude that the observed upregulation of some HSPs in Volkameriana may contribute to increased tolerance to HLB disease.

### Overall metabolism

The repression of key proteins involved in photosynthetic light reactions was linked with the upregulation of starch-related pathways. Infected Volkameriana exhibited up-regulated nitrilases, oxidases, glutathione-S-transferases and other proteins involved in redox state. Infected Volkameriana also exhibited enhanced production of expansins and xyloglucan endotransglycosylases.

Proteins commonly altered by HLB in both genotypes strengthen the data at proteomic level. The WD40 repeat-like protein was upregulated along with some enzymes involved in protein targeting, degradation, and glycosylation such as cysteine peptidase 3, proteinase A1, and cysteine protease. Additional file 5: Figure S3 presents a complete list of commonly regulated proteins and their abundance in the four examined *Citrus* sample categories.

## Conclusions

Forty-six of the 71 proteins were encoded by genes that were found to be transcriptionally regulated by HLB by previous studies in field conditions. Any comparison between transcriptomic and proteomic studies should take into consideration that experiments were performed with different plant material grown under differing environmental, developmental and physiological conditions. We have applied an integrated approach using principal component analysis (PCA), gene set enrichment analysis and functional data mining to identify specific key proteomic changes in response to Huanglongbing disease in these two *Citrus* genotypes. The analysis of post-transcriptional mechanisms is an essential step to link molecular regulatory networks to observed phenotypic changes. Interestingly, the clearest differences between the two genotypes were observed for proteins involved in redox state and detoxification pathways such as glutathione-S-tranferases. HLB disease strongly affected genes and metabolites of the terpenoid, carotenoid, and jasmonic acid pathways [16, 42].

## Methods

### Material and experimental design

Citrus plant materials used in this study were propagated from disease free bud wood obtained from the California Citrus Clonal Protection Program (CCPP). Two-year old Volkameriana (V) (*Citrus* × *volkameriana*) and Navel orange (N) (*Citrus* × *sinensis*) trees were grafted on Carrizo citrange rootstocks (*Citrus sinensis* [L.] Osb. × *Poncirus trifoliata* [L.] Raf.). Trees were grown in pots in the greenhouse under natural light at 17 to 25 °C in the Contained Research Facility (CRF) at UC Davis. Around 10 trees per genotype were infected with CaLas through graft inoculations using a standard inverted “T” budding technique with infected budwood from Lisbon Lemon (*Citrus limon* Burm.f.), with uninocculated trees maintained as an uninfected control. Starting at 3 months after budding, each plant was tested monthly using quantitative RT-PCR for CaLas species as described [43]. Three to four biological replicates of healthy and infected symptomatic trees were chosen for proteomic analysis based on health, phenotype and symptom severity. A pool of five to seven fully expanded leaves at the same developmental stage from each tree was sampled at 8 months, constituting a biological replicate. From infected trees, leaves with characteristic yellowing and blotchy mottled appearance were sampled. Healthy leaves at the same developmental stage were harvested from the uninfected control trees. Petioles from four to six leaves sampled from different parts of each tree were tested by PCR for the presence of CaLas at the time of collection. Midribs and petioles were cut, frozen in liquid nitrogen and stored at −80 °C for protein extraction and iTRAQ analysis. The other parts of the leaves were used to test for pathogen presence. Ct values of infected trees were < 30 while control trees showed no amplified product.

### Protein extraction

Proteins were extracted using a previously described phenol-based procedure [44]. Leaves were ground in a mortar and pestle in liquid nitrogen with 1 % (w/w) PVPP. One hundred mg plant material was resuspended in 600 μL extraction buffer (0.7 M Sucrose, 0.1 M KCl, 0.5 M Tris–HCl pH7.5, 0.5 M EDTA, 1 mM PMSF and 2 % β-mercaptoethanol). Samples were homogenized twice (one min each) with a MM300 TissueLyser (Qiagen). An equal volume of UltraPure Buffer-Saturated Phenol (Invitrogen) was added and the mixture was rehomogenized as described above. After centrifugation at 12,000 × g for 15 min at 4 °C, the upper phenol phase was eliminated and the pellet used for re-extraction in the same buffer. Protein was precipitated from the phenol phase using five volumes saturated ammonium acetate (100 mM) in methanol overnight at −20 °C followed by centrifugation at 12,000 × g for 15 min at 4 °C. Pellets were washed four times with four mL saturated ammonium acetate (100 mM) in methanol and dried 10 min. Proteins were dissolved in urea buffer (7 M urea, 2 M thiourea, 40 mM Tris, 2 % Chaps and 18 mM DTT). The protein concentration was determined using Bradford’s method with BSA as a standard.

### Protein sample preparation and digestion

Samples were precipitated using the ProteoExtract Protein Precipitation Kit (CalBiochem). The resulting protein pellet was solubilized in 400 μL of 50 mM triethyl ammonium bicarbonate (TEAB) and a 100 μL aliquot was taken for digestion. 500 mM tris (2-carboxyethyl)-phosphine (TCEP) (Pierce, Rockford, IL) was added to a final concentration of 10 mM and samples were incubated for 10 min at 90 °C to reduce disulfide bonds. Next, 110 mM iodoacetamide (IAA) was added to a final concentration of 15 mM and incubated for 1 h at room temperature, followed by the addition of 20 μL DTT to quench the IAA reaction. Trypsin (Promega) was next added in a 1:25 ratio (enzyme: protein) and incubated at 37 °C for overnight. The following day, samples were desalted using C18 Macro Spin columns (Nest Group) and dried down by vacuum centrifugation.

### Tandem mass tag labeling

Desalted and lyophilized samples were resuspended in 50 mM TEAB and ~30ug of tryptic digested peptides were taken for TMT labeling. TMT labeling was performed on each aliquot with reporter ions m/z = 126.1, 127.1, 128.1, and 129.1 in 41 μL ethanol, and aliquots were incubated for 60 min at room temperature. 8 μL hydroxylamine 5 % (v:v) was added to quench the reaction and samples were vacuum-centrifuged prior to desalting using C18 Macro Spin columns (Nest Group). Samples were vacuum-centrifuged once more prior to strong cation exchange fractionation.

### SCX fractionation of Pooled TMT-labeled samples

Strong cation exchange (SCX) was carried out using the SCX SpinTips Sample Prep Kit (ProteaBio). Each aliquot was resuspended in 50 μL of the designated buffer and ~10 μg of each sample was pooled prior to SCX fractionation. Samples were fractionated by stepwise addition of 20, 40, 60, 80, 100, 150, 250, and 500 mM ammonium formate in 10 % acetonitrile. All eight fractions, including the initial binding flow through, were vacuum-centrifuged to remove any acetonitrile and desalted using C18 Macro Spin columns (Nest Group).

### LC-MS/MS analysis

LC separation was done on a Waters Nano Acquity UHPLC (Waters Corporation) with a Proxeon nanospray source. Each SCX fraction (9 total) was reconstituted in 2 % acetonitrile /0.1 % trifluoroacetic acid and loaded onto a 100 μm × 25 mm Magic C18 100 Å 5U reverse phase trap. Peptides were eluted using a gradient of 0.1 formic acid (A) and 100 % acetonitrile (B) with a flow rate of 300 nL/min. A 60 min gradient was run with 5 to 35 B over 50 min, 35 to 80 B over 3 min, 80 B for 1 min, 80 to 5 B over 1 min, and finally held at 5 % B for 5 min.

Mass spectra were collected on an Orbitrap Q Exactive Plus mass spectrometer (Thermo Fisher Scientific). A dynamic exclusion of 15 s was used. MS spectra were acquired with a resolution of 70,000 and a target of 1 × 106 ions or a maximum injection time of 30 ms. MS/MS spectra were acquired with a resolution of 17,500 and a target of 5 × 104 ions or a maximum injection time of 50 ms, and a fixed first mass of 110 m/z. Peptide fragmentation was performed using higher-energy collision dissociation with a normalized collision energy value of 30. Unassigned charge states as well as +1 and ions > +5 were excluded from MS/MS fragmentation.

### Data analysis

Tandem mass spectra were extracted and charge states were deconvoluted and deisotoped. All MS/MS samples were analyzed using X! Tandem (The GPM, thegpm.org; version X! Tandem Sledgehammer (2013.09.01.1)). X! Tandem was set up to search the *Citrus sinensis* genome (http://www.ncbi.nlm.nih.gov/protein/?term=txid2706 (March 2014) and the NCBInr citrus database (155,237 entries, March 2014) assuming the digestion enzyme trypsin. X! Tandem was searched with a fragment ion mass tolerance of 20 PPM and a parent ion tolerance of 20 PPM. TMT6plex of lysine and the n-terminus was specified in X! Tandem as a fixed modification.

Scaffold Q+ (version Scaffold_4.4.0, Proteome Software Inc., Portland, OR) was used to quantitate Label Based Quantitation (iTRAQ, TMT, SILAC, etc.) peptide and protein identifications. Peptide identifications were accepted if they could be established at a 99.0 % probability by the Scaffold Local FDR algorithm, which corresponded to a 0.20 spectra decoy FDR and a 5.0 % protein decoy FDR with 1 identified peptides per protein. Protein probabilities were assigned by the Protein Prophet algorithm [45]. Proteins that contained similar peptides and could not be differentiated based on MS/MS analysis alone were grouped to satisfy the principles of parsimony. Proteins sharing significant peptide evidence were grouped into clusters. Proteins sharing significant peptide evidence were grouped into clusters according to the algorithm described in i-Tracker [46]. Individual quantitative samples were normalized within each acquisition run. Intensities for each peptide identification were normalized within the assigned protein. All normalization calculations were performed using medians to multiplicatively normalize data. Differentially expressed proteins were determined using Permutation Test analysis.

### Statistical and functional data mining

Scaffold 4 was used to perform the first functional, annotation and quantitative analysis of the proteomic data. Arabidopsis orthologs, annotations, unique peptides and spectrum counts, and normalized quantitative values were determined for each sequenced and identified peptide. Data were blasted against the *Citrus x sinensis* (L.) and *Candidatus* liberibacter asiaticus (strain psy62) genomes. Arabidopsis orthologs were determined for each sequenced peptide by blastx (e-value < 10–4) to the TAIR database of predicted proteins in Arabidopsis (TAIR10_- pep_20101028; [47]). Blastx output was processed using custom scripts to calculate the best correspondence between individual citrus peptide sequences and Arabidopsis proteins, based on alignments over the entire length of each sequence. Lists of significantly differentially expressed proteins (*p* < 0.05, absolute value of log2 fold change > 0.5 or < − 0.5) were determined in pairwise comparison (infected/healthy) for each genotypes. This statistical analysis was performed using MeV software. Functions of differentially regulated proteins (as Arabidopsis orthologs) were visualized using MapMan [48]. Gene set enrichment analysis was performed using DAVID Bioinformatics resource 6.7 based on KEGG maps. The corresponding Arabidopsis orthologs of each protein upregulated or downregulated during infection for each genotype was loaded as a gene list in DAVID (*p* < 0.05). Arabidopsis orthologs were determined for each citrus protein and the gene set enrichment analysis was obtained comparing the list of differentially regulated proteins with all those identified by the proteomic analysis.

The PageMan visualization tool was used for GSEA with the Wilcoxon test (no correction and 1.0 as ORA cutoff). Principal component analysis (PCA) was performed using SAS II (2008) SAS/STAT software (SAS Institute). Principal component analysis was applied to the ratio matrix of gene expression data to examine the contribution of each target gene to the separation of sample classes. A biplot was constructed based on the first two principal components.

## Abbreviations

2-DE, Two-dimensional electrophoresis; ABC transporters, ATP-binding cassette transporters; ADPase, ADP glucose-pyrophospholyase; BAK1, BCL2-Antagonist/Killer 1; CaLas, *Candidatus* Liberibacter asiaticus; CCPP, Citrus Clonal Protection Program; CRF, Contained Research Facility; CYP711A1, cytochrome P450, family 711, subfamily A, polypeptide 1; DMAPP, Dimethylallyl pyrophosphate; DUF26, Domain of Unknown Function 26; ET, Ethylene; GASA1, GAST1 protein homolog 1; GPT2, Glucose-phosphate-transporter 2; GSTS, Glutathione S-transferases; HLB, Huanglongbing; HSP, Heat shock proteins; IAA, Indole 3 acetic acid; ICP-MS, Inductively coupled plasma mass spectrometry; IPP, Isopentenyl pyrophosphate; iTRAQ, Isobaric tags for relative and absolute quantitation; JA, Jasmonic acid; LRR, Leucine rich repeats; MVA, mevalonate pathway; N, Navel orange; NBS-LRR, Nucleotide-binding site–leucine-rich repeat; PAL, Phenylammonialyase; PAMP, Pathogen-associated molecular patterns; PCA, Principal component analysis; PEP, phospho-enol-pyruvate; PPM, Parts-per-million; RHM1, UDP-L-rhamnose synthase; RLKs, receptor-like kinases; SA, Salicilic acid; SAUR, small auxin-up RNA; SCX, Strong cation exchange; TCA cycle, Tricarboxylic acid cycle; TCEP, Tris (2-carboxyethyl)-phosphine; TCH4, xyloglucanxyloglucosyltransferase; TEAB, Triethyl ammonium bicarbonate; TMT labeling, Tandem mass tags; UXS2, UDP-glucuronic acid decarboxylase 2; UXS6, UDP-glucuronic acid decarboxylase 6; V, Volkameriana

## Additional files

Additional file 1: Table S1. Differentially regulated host proteins in control and infected leaf tissues of Navel orange (p-value < 0.05). Arabidopsis orthologs and log fold ratio are indicated. (XLS 67 kb) Additional file 2: Table S2. Differentially regulated host proteins in control and infected leaf tissues of Volkameriana (p-value < 0.05). Arabidopsis orthologs and log fold ratio are indicated. (XLS 55 kb) Additional file 3: Figure S1. Metabolism oveview of proteomic changes in response to HLB disease, comparing effects in the two Citrus genotypes. (PDF 115 kb) Additional file 4: Figure S2. HLB-differentially regulated proteins involved in secondary metabolism in the two Citrus genotypes. Each colored square represents the expression change (see color key) in a protein associated with the biosynthetic pathway. (TIF 268 kb) Additional file 5: Figure S3. A list of commonly regulated proteins by HLB, comparing effects in the two Citrus genotypes. VC = Volkameriana control (healthy), VI = Volkameriana Infected, NC = Navel orange control (healthy), NI = Navel orange infected. Annotation and Citrus ID are indicated. (PDF 121 kb)
