# Supplementary material for: Proteomic analysis highlights the role of detoxification pathways in increased tolerance to Huanglongbing disease
Source: BMC Plant Biol. 2016 Jul 28;16:167. doi: 10.1186/s12870-016-0858-5 (PMC4963945; doi:10.1186/s12870-016-0858-5)
Supplement: Additional file 3: Figure S1. — Metabolism oveview of proteomic changes in response to HLB disease, comparing effects in the two Citrus genotypes. (PDF 115 kb) [file 12870_2016_858_MOESM3_ESM.pdf]

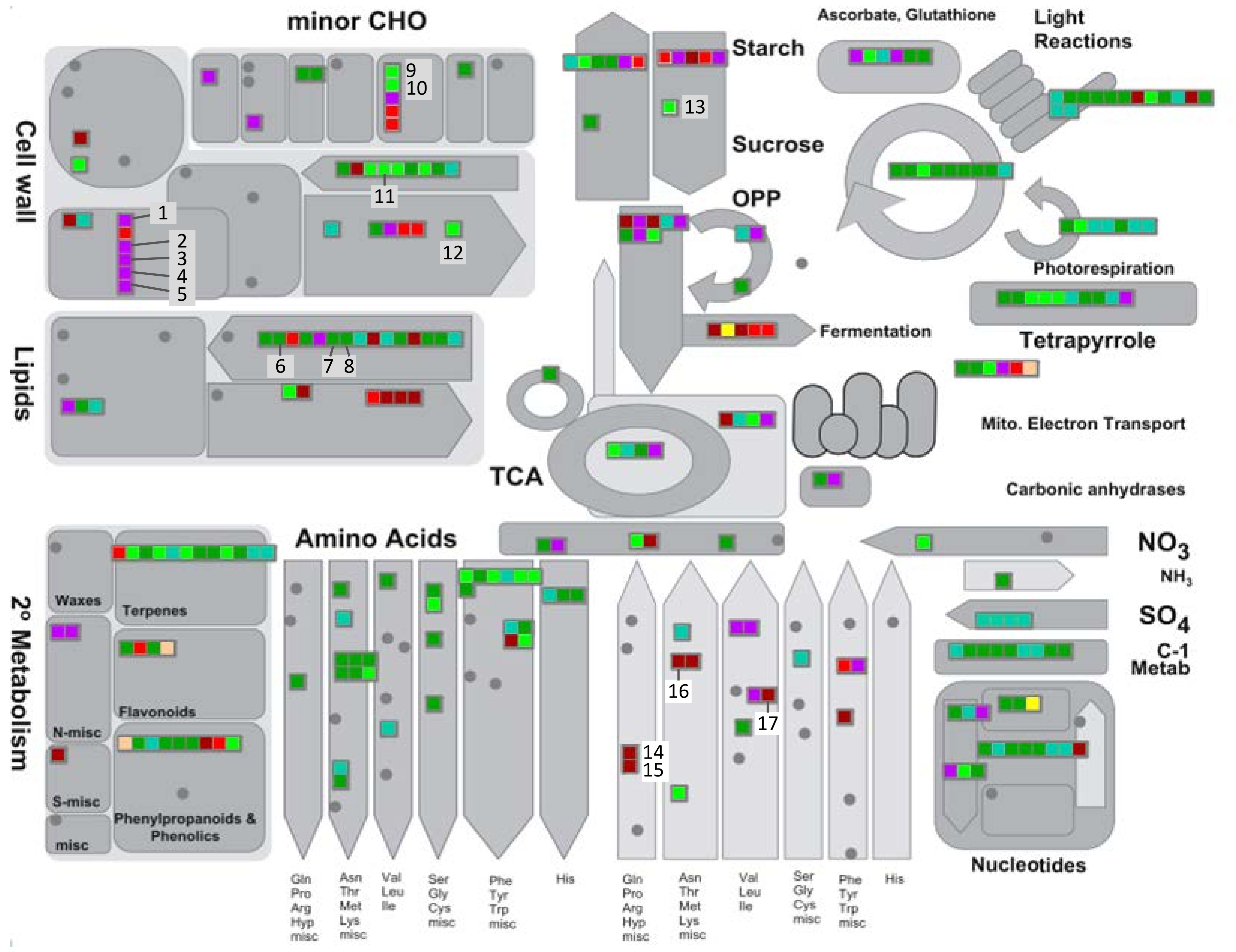


1) Expansin A1
2) Expansin A8
3) Expansin like B1
4) Xyloglucan endotransglycosyl. 6
5) Xyloglucan-glucosyltransf. (TCH4)
6) Acetyl-CoA carboxylase
7) 3-oxoacyl reductase
8) Enoyl-(acyl-carrier protein)-reductase
9) Aldo/keto reductase 1
10) Aldo/keto reductase 2
11) UDP-glucoronate decarboxylase
12) Pectase lyase
13) Sucrose synthase 4
14) Arginase
15) Pyrroline-5-carboxylase reductase
16) Lactoylglutathione lyase
17) 3-hydroxylmethylglutaryl-CoA lyase

Upregulated
- Navel orange
- Volkameriana
- both genotypes

Downregulated
- Navel orange
- Volkameriana
- both genotypes

- Upregulated in navel orange, down in Volkameriana
- Upregulated in Volkameriana, down in navel orange
