# Supplementary figures and images for: Proteomic analysis highlights the role of detoxification pathways in increased tolerance to Huanglongbing disease
Source: BMC Plant Biol. 2016 Jul 28;16:167. doi: 10.1186/s12870-016-0858-5 (PMC4963945; doi:10.1186/s12870-016-0858-5)

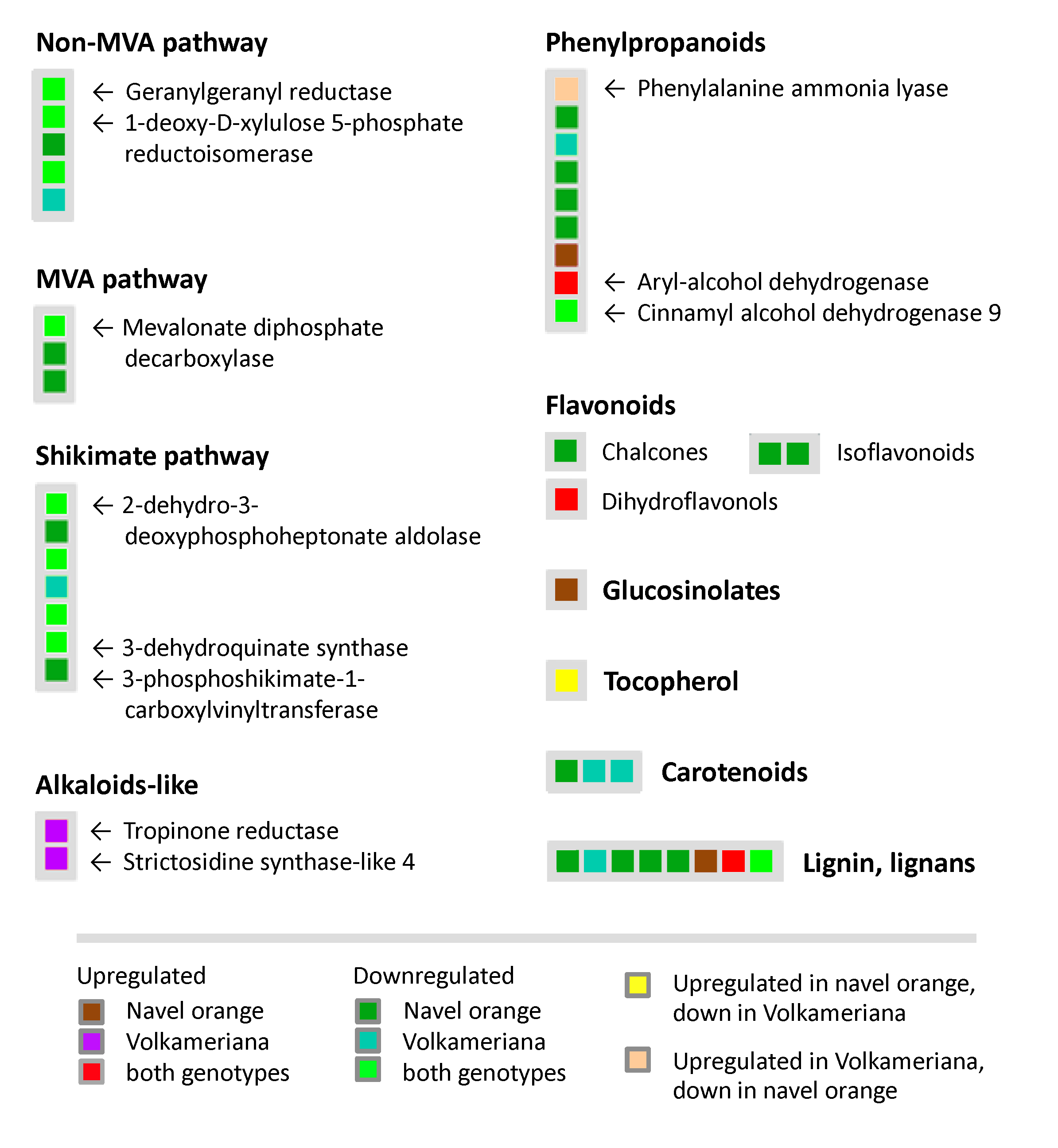

Supplement: Additional file 4: Figure S2. — HLB-differentially regulated proteins involved in secondary metabolism in the two Citrus genotypes. Each colored square represents the expression change (see color key) in a protein associated with the biosynthetic pathway. (TIF 268 kb) [file 12870_2016_858_MOESM4_ESM.tif]
