# Supplementary material for: Proteomic analysis highlights the role of detoxification pathways in increased tolerance to Huanglongbing disease
Source: BMC Plant Biol. 2016 Jul 28;16:167. doi: 10.1186/s12870-016-0858-5 (PMC4963945; doi:10.1186/s12870-016-0858-5)
Supplement: Additional file 5: Figure S3. — A list of commonly regulated proteins by HLB, comparing effects in the two Citrus genotypes. VC = Volkameriana control (healthy), VI = Volkameriana Infected, NC = Navel orange control (healthy), NI = Navel orange infected. Annotation and Citrus ID are indicated. (PDF 121 kb) [file 12870_2016_858_MOESM5_ESM.pdf]

Treatments	Protein function/name	Accession
RNA regulation of transcription/RNA processing		
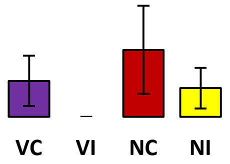	GATA type zinc finger transcription factor family protein	orange1.1g029070m<br>orange1.1g029735m
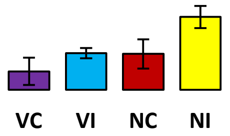	Transducin/WD40 repeat-like superfamily protein	orange1.1g039790m
**Protein targeting/Protein degradation/Protein glycosylation**		
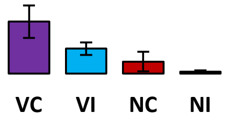	Translocon at the inner envelope membrane of chloroplasts 110	orange1.1g001818m<br>orange1.1g003418m
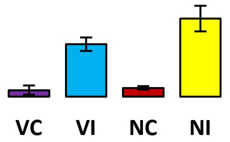	Xylem bark cysteine peptidase 3	orange1.1g014761m<br>orange1.1g019447m
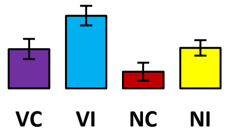	Aspartic proteinase A1	orange1.1g010466m<br>orange1.1g018226m<br>orange1.1g010486m<br>orange1.1g016523m<br>orange1.1g022584m<br>orange1.1g015145m
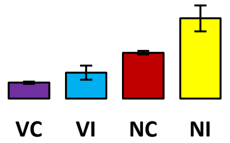	Granulin repeat cysteine protease family protein	orange1.1g024783m
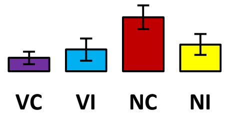	Regulatory particle triple-A 1A	orange1.1g014665m<br>orange1.1g015875m
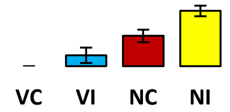	Vacuolar processing enzyme beta	orange1.1g021650m<br>orange1.1g023458m
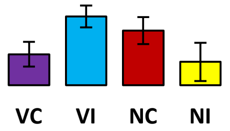	Ribophorin I	orange1.1g007137m

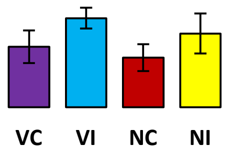	20S proteasome alpha subunit E2	orange1.1g042723m<br>orange1.1g030843m
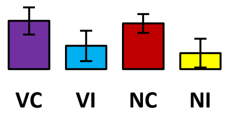	Hydroxyproline-rich glycoprotein family protein	orange1.1g016002m<br>orange1.1g020409m<br>orange1.1g022173m
	Amino acid metabolism	
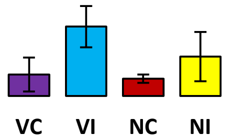	Putative fumarylacetoacetase	orange1.1g018339m<br>orange1.1g019941m<br>orange1.1g012786m<br>orange1.1g022156m<br>orange1.1g014784m
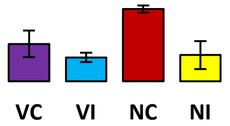	Putative chorismate synthase / 5-enolpyruvylshikimate-3-phosphate phospholyase	orange1.1g022731m
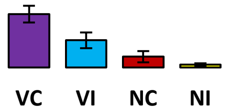	Aspartate aminotransferase	orange1.1g012095m<br>orange1.1g018665m
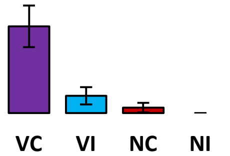	S-adenosylmethionine synthetase 2	orange1.1g044843m
	Stress biotic	
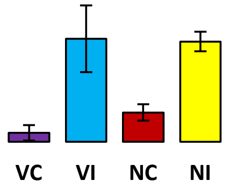	Kunitz family trypsin and protease inhibitor protein	orange1.1g027419m<br>orange1.1g030834m<br>orange1.1g048141m<br>orange1.1g038462m<br>orange1.1g028238m<br>orange1.1g031474m<br>orange1.1g040351m<br>orange1.1g026834m
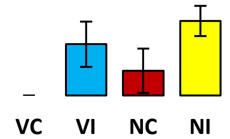	Chlorophyllase	orange1.1g020188m
	Hormone metabolism	
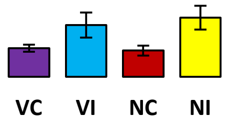	NAD(P)-linked oxidoreductase superfamily protein	orange1.1g019203m

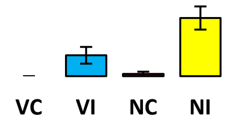	Lipoxygenase 2	orange1.1g002649m<br>orange1.1g002672m<br>orange1.1g002644m<br>orange1.1g002706m<br>orange1.1g002670m<br>orange1.1g007674m<br>orange1.1g004307m<br>orange1.1g006999m
Secondary metabolism		
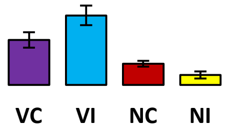	NAD(P)-binding Rossmann-fold superfamily protein	orange1.1g025971m<br>orange1.1g020110m
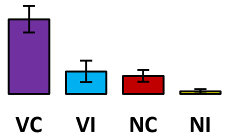	1-deoxy-D-xylulose 5-phosphate reductoisomerase	orange1.1g012004m<br>orange1.1g013846m
Other		
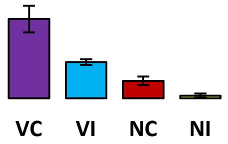	Coproporphyrinogen III oxidase<br>(tetrapyrrole synthesis)	orange1.1g016102m
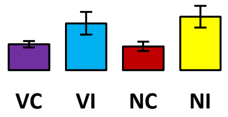	NAD(P)-linked oxidoreductase superfamily protein<br>(minor CHO metabolism)	orange1.1g017732m<br>orange1.1g020098m<br>orange1.1g023567m<br>orange1.1g018530m
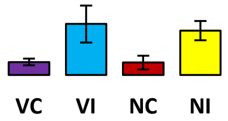	Calreticulin 3 (signalling)	orange1.1g014968m<br>orange1.1g015060m<br>orange1.1g020827m
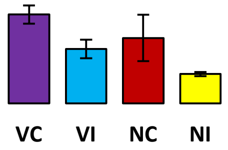	Glyceraldehyde-3-phosphate dehydrogenase B subunit<br>(calvin cycle)	orange1.1g012940m<br>orange1.1g025793m<br>orange1.1g015291m<br>orange1.1g021979m
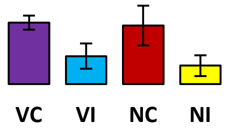	Tubulin alpha-2 chain (cell organisation)	orange1.1g013066m<br>orange1.1g014077m<br>orange1.1g037616m
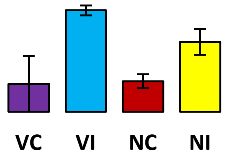	Alcohol dehydrogenase 1 (fermentation)	orange1.1g047713m<br>orange1.1g035170m<br>orange1.1g017336m<br>orange1.1g016933m<br>orange1.1g019414m

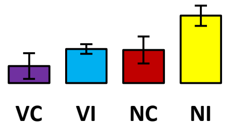	Transducin family protein / WD-40 repeat family protein (development)	orange1.1g010997m<br>orange1.1g016547m<br>orange1.1g046899m<br>orange1.1g021657m
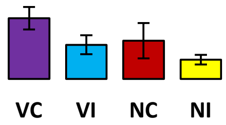	ATPase F1 complex, gamma subunit protein (lightreaction)	orange1.1g017613m
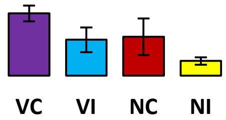	Glutamate synthase 1 (N-metabolism)	orange1.1g000461m
	Miscellaneous	
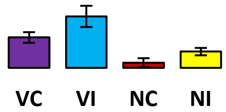	D-mannose binding lectin protein with Apple-like carbohydrate-binding domain	orange1.1g048464m<br>orange1.1g041743m
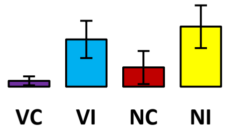	O-Glycosyl hydrolases family 17 protein	orange1.1g019362m
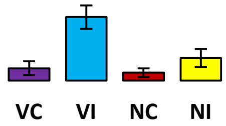	Glutathione s-transferase tau 7	orange1.1g027333m<br>orange1.1g027498m<br>orange1.1g043218m
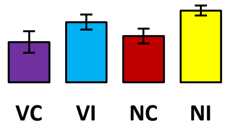	Alpha/beta-Hydrolases superfamily protein	orange1.1g023687m<br>orange1.1g031524m
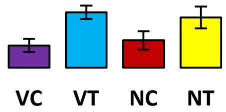	Zinc-binding dehydrogenase family protein	orange1.1g037444m<br>orange1.1g039200m<br>orange1.1g018404m
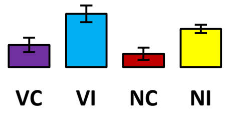	Zinc-binding dehydrogenase family protein	orange1.1g019042m<br>orange1.1g042426m<br>orange1.1g033914m<br>orange1.1g026217m<br>orange1.1g019332m<br>orange1.1g019291m
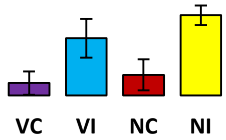	Glycosyl hydrolase family 38 protein	orange1.1g015028m<br>orange1.1g011547m

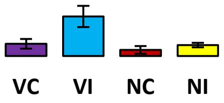	Glycosyl hydrolase family 38 protein	orange1.1g002036m<br>orange1.1g002170m<br>orange1.1g002764m
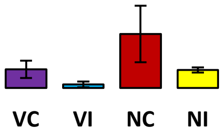	Pectin methylesterase inhibitor superfamily protein	orange1.1g029238m<br>orange1.1g027094m
	Unknown	
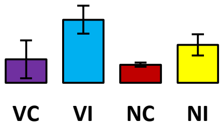	DC1 domain-containing protein	orange1.1g008336m<br>orange1.1g011791m<br>orange1.1g013684m<br>orange1.1g021494m<br>orange1.1g014225m<br>orange1.1g015833m
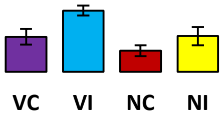	Class I glutamine amidotransferase-like superfamily protein	orange1.1g016237m<br>orange1.1g029299m
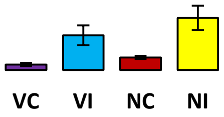	Thioredoxin family protein	orange1.1g024026m<br>orange1.1g023026m
